# Antioxidant Capacities and Enzymatic Inhibitory Effects of Different Solvent Fractions and Major Flavones from Celery Seeds Produced in Different Geographic Areas in China

**DOI:** 10.3390/antiox11081542

**Published:** 2022-08-09

**Authors:** Chao Zhang, Jing Yu, Qiang Tu, Fu Yan, Zhao Hu, Youming Zhang, Chun Song

**Affiliations:** 1 Institute of Marine and Technology, Shandong University, 72 Binhai Road, Jimo, Qingdao 266237, China; 2 State Key Laboratory of Microbial Technology, Shandong University, 72 Binhai Road, Jimo, Qingdao 266237, China; 3 Department of Nephrology, Qilu Hospital Dezhou Hospital, Shandong University, Dezhou 253000, China

**Keywords:** Celery (*Apium graveolens* L.) seed, different solvent fractions, main flavone glycosides, antioxidant capacities, α-amylase inhibitory activities, α-glucosidase inhibitory activities, molecular docking

## Abstract

To extend the application of celery (*Apium graveolens* L.) seeds, the antioxidant and enzymatic inhibitory activities of different fractions and their main flavones were investigated. The n-butanol fractions possessed the highest total phenolic content (TPC) and total flavonoid content (TFC) values. The n-butanol fractions from Northeast China samples exhibited the strongest free radical scavenging (DPPH IC_50_ = 20.27 μg/mL, ABTS IC_50_ = 15.11 μg/mL) and ferric reducing antioxidant power (FRAP 547.93 mg trolox (TE)/g) capacity, while those collected from Hubei China showed the optimal cupric reducing antioxidant capacity (CUPRAC) values (465.78 mg TE/g). In addition, the dichloromethane fractions from Jiangsu samples displayed a maximum Fe^2+^ chelating capacity (20.81 mg ethylene diamine tetraacetic acid (EDTA)/g). Enzyme level experiments indicated polyphenolic compounds might be the main hypoglycemic active components. Subsequently, the enzyme inhibitory activity of nine main flavones was evaluated. Chrysoeriol-7-O-glucoside showed better α-glucosidase inhibitory activity than others. However, apigenin showed the best inhibitory effect on α-amylases, while the presence of glycosides would reduce its inhibitory effect. This study is the first scientific report on the enzymatic inhibitory activity, molecular docking, and antioxidant capacity of celery seed constituents, providing a basis for treating or preventing oxidative stress-related diseases and hyperglycemia.

## 1. Introduction

Diabetes mellitus (DM) is a class of diseases related to metabolic disorders, and its morbidity and mortality are increasing yearly [1]. Chronic hyperglycemia in DM leads to severe complications such as diabetic ophthalmopathy, nephropathy, hypertension, angiocardiopathy, neuropathy, and diabetic foot, which are severe threats to human life and health [2]. Many studies have demonstrated the close relationship between hyperglycemia, oxidative stress, and diabetic complications [3]. In addition, there is a crosstalk between the causes of increased free radical production in organisms and the factors that trigger DM [4]. Unfortunately, there is no cure for DM, but it can be treated through DM education, dietary treatment, exercise therapy, pharmacotherapy, glucose monitoring, and the detection and control of other cardiovascular disease risk factors [5,6]. Most clinical drugs used to control DM, such as acarbose, metformin, or miglitol, have many side effects, including gastrointestinal disturbance, weight gain, hypoglycemia, edema, and liver damage [7]. Therefore, nutritionists and medical researchers are more critical in developing natural hypoglycemic and antioxidant stress products with higher safety factors.

In recent years, polyphenolic foods rich in antioxidants have received significant attention worldwide for their potential health benefits. Flavonoids are primary polyphenolic compounds in various herbs and fruits, such as hawthorn and pomegranate [8,9]. Studies have found that the inhibitory effects of α-glucosidase and α-amylase were directly related to the position and number of hydroxyl groups of flavonoids [10,11]. Flavonoids can also indirectly lower postprandial blood glucose by inhibiting dipeptidyl peptidase-IV (DPP-IV) activity and reducing the inactivation of glucagon-like peptide-1 (GLP-1) and gastric inhibitory polypeptide (GIP) [12]. In addition, polyphenolic compounds can reduce starch digestibility by forming complexes with starch through hydrophobic interactions [13]. More importantly, polyphenolic compounds are excellent free radical scavengers that can delay or inhibit cellular damage through free radical scavenging properties, thereby delaying oxidative stress [14].

Celery is an annual or perennial umbellifer plant that grows throughout Europe and tropical and subtropical regions of Africa and Asia [15]. Celery is also termed “kitchen medicine“ or “medicinal celery“ since its roots, stems, leaves, and seeds are all available for medicinal use. Modern pharmacological studies have shown that celery has hypotensive and hypolipidemic effects [16]. Furthermore, celery seeds can lower blood pressure and lipid levels in the short term without toxic side effects because they contain 50 times more antihypertensive and lipid-lowering ingredients than other parts of celery [16]. Celery seeds are also used in the Uyghur medicine of China to treat hypertension, arthritis, rheumatoid arthritis, and kidney diseases.

**Figure 1:**
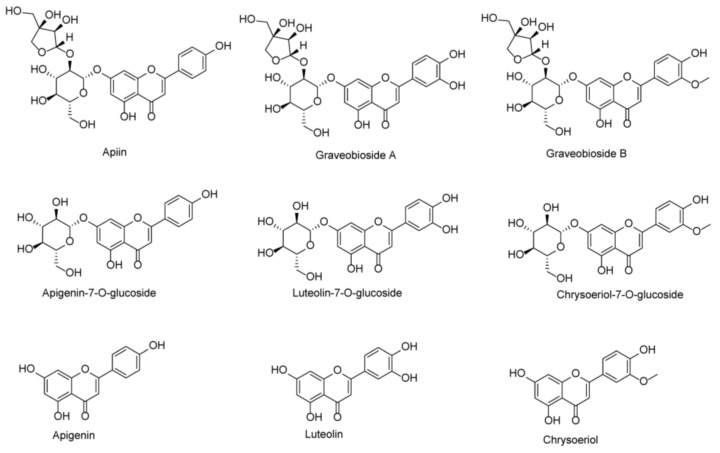
Chemical structures of apiin, graveobioside A, graveobioside B, apigenin-7-O-glucoside, luteolin-7-O-glucoside, chrysoeriol-7-O-glucoside, apigenin, luteolin, and chrysoeriol.

Numerous flavones have been isolated from celery seeds, including luteolin, apigenin, corresponding glycosides, etc. (Figure 1) [17,18]. We previously identified and isolated three major flavone glycosides, graveobioside A, graveobioside B, and apiin, from the ethanolic crude extracts of Northeast China samples using different analytical methods (submitted in other articles). Many reports have found that different geographical locations and environments can result in different types and levels of polyphenolic compounds [19,20]. Therefore, the content and structure of flavone components in celery seeds from different geographical regions may differ, resulting in different antioxidant and enzyme inhibiting effects. Flavones of celery seeds, such as luteolin, have been reported to show prominent α-glucosidase and α-amylase inhibitory activities and can be used as a possible efficient alternative approach to treating DM [21]. Besides, the flavones of celery seeds are excellent natural antioxidants that can prevent or treat oxidative stress-related diseases [14].

To date, the TPC, TFC, antioxidant, α-glucosidase inhibitory, and α-amylase inhibitory properties of celery seeds from different geographical regions of China are unknown. Therefore, we selected five celery seed samples from different geographical regions of China based on external factors such as specific growing areas, average temperature, and sunlight duration and extracted flavones to characterize them by high-performance liquid chromatography-time-of-flight-mass spectrometry (HPLC-TOF-MS). Subsequently, the TPC, TFC, antioxidant, α-glucosidase inhibitory, and α-amylase inhibitory activities of varying solvent fractions and main flavones in celery seeds were systematically investigated. Moreover, the molecular docking studies between α-glucosidase with different flavones were also conducted. This study provides the first scientific report on the α-glucosidase and α-amylase inhibitory activity and antioxidant capacity of celery seed components, providing a scientific basis for the treatment or prevention of oxidative stress-related diseases and hyperglycemia.

## 2. Materials and Methods

### 2.1. Samples

This study was carried out on five *celery seed* samples in July 2019 that came from different geographical areas of China (1 sample from the Shandong province (East China), 1 sample from the Guangxi province (South China), 1 sample from the Northeast (Northeast China), 1 sample from the Jiangsu province (East China), and 1 sample from the Hubei province (Central China)). All samples were deposited in the dark until used at the state key laboratory of microbial technology of Shandong University (Qingdao, Shandong, China).

### 2.2. Reagents

2,2′-Azino-bis(3-ethyl-benzothiazoline-6-sulfonic acid) (ABTS) and 2,2-diphenyl-1-picryhydrazyl (DPPH), Folin-Ciocalteu reagent, potassium persulfate, 2,4,6-tris(2-pyridyl)-s-triazine (TPTZ), trolox, rutin, gallic acid, ferrouschloride (FeCl_3_), ferrozine, cupric chloride (CuCl_2_), neocuproine, ascorbic acid (VC), aluminum chloride (AlCl_3_), 4-nitrophenyl α-glucopyranoside (PNPG), and acarbose were obtained from Macklin (Shanghai, China). Butylated hydroxytoluene (BHT) and ethylene diamine tetraacetic acid (EDTA) were purchased from Sigma-Aldrich (Shanghai, China). Saccharomyces cerevisiae α-glucosidase, porcine pancreatic α-amylase, apigenin-7-O-glucopyranoside, chrysoeriol-7-O-glucosid, and luteolin-7-O-glucoside were purchased from Shanghai Yuanye Biotechnology Co. (Shanghai, China). Methanol (MeOH), ethanol (EtOH), and dichloromethane (DCM) were purchased from Tianjin Fuyu (Tianjin, China). Ammonium acetate (NH_4_Ac), iodine (I_2_), potassium iodide (KI), soluble-starch, acetic acid (AcOH), n-butanol (nBuOH), and silica sand were obtained from Guoyao (Shanghai, China). The products were purified by flash column chromatography on 200–300 mesh silica gel purchased from Xinnuo Huagong (Yantai, Shandong, China). Methanol and acetonitrile of the high-performance liquid chromatography (HPLC) grade were purchased from Thermo Fisher Scientific (Waltham, MA, USA). DMSO-D6 was purchased from J&K (Beijing, China).

Apiin, graveobioside A, graveobioside B, apigenin, luteolin, and chrysoeriol were isolated from ethanol extracts of the Northeast China celery seeds (submitted in the other manuscript).

### 2.3. Preparation of Celery Seed Extracts

A total of 60 g of celery seed powder (the volatile oil was removed) was extracted with EtOH at reflux for 1.5 h three times and combined with the filtrates. The EtOH was removed by concentration under reduced pressure (IKA RV10, Breisgau, Germany), and the filtrate was pulped with DCM. The DCM layer and the solids were collected. Subsequently, the solid was dissolved in water and extracted with nBuOH to obtain the nBuOH layer and aqueous layer. Then, concentration afforded different fractions of celery seeds.

### 2.4. Flavones Analysis by HPLC-TOF-MS

The flavones in the nBuOH fractions of celery seeds were analyzed and characterized by HPLC-TOF-MS (Bruker 40000R, Bremen, Germany). The preferred wavelengths for the flavone glycosides were 270 nm. The chromatographic column employed in the experiments was a Shimadzu C-18 (4.6 × 150 mm, 5 μm) column. Gradient separation using 0.1% formic acid in water (*v*/*v*) (solvent A) and acetonitrile (solvent B) as mobile phase was as follows: 0–10 min, linear gradient from 1 to 15% B; 10–30 min, linear gradient from 15 to 30% B; 30–45 min, linear gradient from 30 to 45%; 45–50 min, 45% B; 50–53 back to initial conditions at 1% B; and 53–60 min, at 1% B, and the solvents used were all HPLC grades. The flow rate was set at 1 mL/min. The column temperature was 30 °C, and 10 μL was injected into the Bruker HPLC system. The flavones were identified based on retention times, UV-Vis spectra, mass spectra, and literature data.

### 2.5. Quantitative Analysis of Flavones in the nBuOH Fraction by HPLC-UV

The standard curves of apiin, graveobioside A, graveobioside B, apigenin-7-O-glucopyranoside, chrysoeriol-7-O-glucosid, and coefficients are shown in Figure S2A–F.

The preferred wavelengths were 270 nm. The chromatographic column employed in the experiments was a Shimadzu C-18 (4.6 × 150 mm, 5 μm) column. Gradient separation using 0.1% formic acid in water (*v*/*v*) (solvent A) and acetonitrile (solvent B) as mobile phase was as follows: 0–10 min, linear gradient from 1 to 15% B; 10–30 min, linear gradient from 15 to 30% B; 30–45 min, linear gradient from 30 to 45%; 45–50 min, 45% B; 50–53 back to initial conditions at 1% B; and 53–60 min, at 1% B, and the solvents used were all HPLC grades. The flow rate was set at 1 mL/min. The column temperature was 30 °C, and 10 μL samples (80 μg/mL) were injected into the HPLC system (SHIMADZU LC-20AT, Kyoto, Japan).

### 2.6. Measurement of Total Phenolic Content (TPC)

TPC was measured using Folin-Ciocalteau’s method with slight modifications [22]. Shortly after, 0.25 N Folin-Ciocalteau’s reagent (50 μL) was added to a 96-well microplate, then each fraction or gallic acid (50 μL) was mixed. The mixture was incubated for 5 min. Subsequently, 5% Na_2_CO_3_ solution (100 μL) was added and reacted for another 15 min. The absorbance was recorded at 750 nm (SpectraMax M5, Molecular Devices, Sunnyvale, CA, USA). The TPC of the extracts was determined from a standard calibration curve using gallic acid in the range of 0–30 μg/mL with an R^2^ value of 0.9976 (Figure S2G). The TPC was calculated as milligrams of gallic acid equivalent (GAE)/g dry extract.

### 2.7. Measurement of Total Flavonoid Content (TFC)

The TFC was measured using the aluminum chloride colorimetry method with slight modifications [9]. Briefly, 100 μL of sample solution and 100 μL 2% AlCl_3_ solution were added for 15 min. The absorbance was measured at 420 nm. The TFC of the extracts was determined from a standard calibration curve using quercetin in the range of 0–50 μg/mL with an R^2^ value of 0.9995 (Figure S2H). Total flavonoid contents were then calculated as milligrams of rutin equivalent (RE)/g dry extract.

### 2.8. DPPH Radical Scavenging Assay

The scavenging ability of the free radical DPPH was investigated as described with slight modification [22]. The sample solution (100 μL) and DPPH reagent (0.11 mg/mL, 100 μL) were mixed and incubated (30 min) at room temperature and in darkness. Then, a multi-detection microplate reader was used to measure the absorbance at 517 nm (As). The control group was mixed with DMSO and DPPH reagent in the same volume (A_0_). A formula was used to calculate the DPPH radical scavenging activity:

DPPH radical scavenging activity (%) = [(A_0_ − As)/A_0_] × 100,

where A_0_ is the absorbance of the control (untreated group), and As is the absorbance of the test sample. BHT and VC were used as the positive control.

### 2.9. ABTS Radical Scavenging Assay

The ABTS radical scavenging activity of each fraction was measured based on the method with slight modifications. A 28 mM ABTS and a 9.6 mM potassium persulfate working solution were prepared, mixed in equal volumes, and the mixture was left to stand for 16 h at room temperature, protected from light. The working solution was diluted with EtOH to reach an absorbance of 0.70 ± 0.02 at 740 nm and used for further experiments. Different extracts or compounds (20 μL) concentrations were mixed with the working solution (180 μL). The mixture was incubated at room temperature for 15 min, and the absorbance was recorded at 734 nm (As). DMSO and ABTS reagents in the same volume were mixed to measure the absorbance as the control (A_0_). Then, the antioxidant activity of the mixture was determined by calculating the decrease using the following formula:

ABTS radical inhibiting activity (%) = (A_0_ − As)/A_0_ × 100,

where A_0_ is the absorbance of the control (untreated group), and As is the absorbance of the test sample. BHT and VC were used as the positive control.

### 2.10. Ferric Reducing Antioxidant Power (FRAP)

The measurement of FRAP was based on previous studies with slight modifications [23]. A total of 180 uL of FRAP working solution (acetate buffer (pH 3.6): ferric chloride solution (20 mM): TPTZ solution (10 mM) = 10:1:1) was mixed with 20 μL of sample solution and incubated for 30 min at room temperature. The absorbance was measured at 593 nm. The standard curve was linear from 0 to 30 μg Trolox with an R^2^ value of 0.9999 (Absorbance: y = 0.0943x + 0.0126, Figure S2I). The results are expressed as milligrams of TE/g dry weight.

### 2.11. Cupric Reducing Antioxidant Capacity (CUPRAC)

The cupric ion reducing antioxidant capacity (CUPRAC) was measured according to the method of Drouet et al. with modifications [24]. Briefly, 50 μL of the extract sample was mixed with 150 μL of the CUPRAC solution (composed of 10 mM CuCl_2_, 7.5 mM neocuproine, and 1 M NH_4_Ac buffer pH 7; ratio 1:1:1 (*v*/*v*/*v*)). After incubation for 15 min at room temperature, the absorbance value at 450 nm of the reaction mixture was measured. The results were reported as millimoles of Trolox equivalents (TE) per gram of extract (mg TE/g extract) (calibration curve from different concentrations: 0–70 μg/mL; Absorbance: y = 0.0279x + 0.0126, R² = 0.9997, Figure S2J).

### 2.12. Metal Chelating Activity on Ferrous Ions

The iron-chelating activity of each fraction was estimated by the method described by Sapatinha et al. with modifications [25]. Briefly, 50 μL of each sample solution and 50 μL of 1 mM FeCl_2_ were mixed. Then, the reaction was initiated by adding 100 μL of 2.5 mM ferrozine solution. The mixture was kept at room temperature for 10 min. The absorbance of the resulting solution was measured at 562 nm. The results are expressed as milligrams of ethylenediaminetetraacetic acid (EDTA) equivalent per gram of extract (mg EDTAE/g extract) (calibration curve from different concentrations: 0–55 μg/mL; Absorbance: y = −0.0477x + 3.0616, R² = 0.9966, Figure S2K).

### 2.13. α-Glucosidase Inhibitory Activity Assay

The α-glucosidase inhibition test was performed as previously described with some variations [26]. PBS (0.1 M, pH = 6.8), α-glucosidase (5 U/mL), and PNPG (5 mM) were prepared and stored at 4 °C for further use. In total, 20 μL of different concentration sample solution (DMSO dissolved) or DMSO, 60 μL of PBS, and 20 μL of α-Glucosidase (0.5 U/mL) were added to a 96-well plate and incubated for 20 min at 37 °C. Then, 20 μL of PNPG (5 mM) solution was added and incubated for another 15 min at 37 °C. Subsequently, 80 μL of Na_2_CO_3_ (0.1 M) solution was added to terminate the reaction. After the reaction, the absorbance of each well was measured using a microplate reader (SpectraMax M5, Molecular Devices, Sunnyvale, CA, USA) at 405 nm. Inhibition (%) was calculated based on absorbance with the following formula: Inhibitor (%) = (A_0_ − As)/A_0_ × 100%, where A_0_ is the absorbance of the blank group, and As is the absorbance of the test group. The IC_50_ values were calculated using GraphPad Prism 8.0 (GraphPad Software, Inc., La Jolla, CA, USA) to evaluate the α-glucosidase inhibition activity of the tested compounds.

### 2.14. α-Amylase Inhibitory Activity

The inhibition assay of α-amylase was conducted using the conditions previously reported with slight modifications [27]. PBS (0.1 M, pH = 6.8), α-amylase (10 U/mL), and 0.2% soluble starch were prepared and stored at 4 °C for further use. Different concentrations of extracts or compounds (DMSO dissolved) or DMSO (20 μL) were mixed with α-amylase solution (60 μL, 1 U/mL) in a 96-well plate and incubated for 20 min at 37 °C. Subsequently, 0.2% soluble starch (20 μL), the substrate, was added and incubated for another 15 min in dry baths at 37 °C. Subsequently, the reaction was terminated after adding 1 M HCl solution (20 μL). Finally, 80 μL iodine reagent solution (1.0 g iodine and 8.0 g potassium iodide dissolved in 100 mL water) was added to the mixture and the absorbance was taken at 620 nm (SpectraMax M5, Sunnyvale, CA, USA). Inhibition (%) was calculated based on absorbance with the following formula: Inhibition (%) = (A_0_ − As)/A_0_ × 100%, where A_0_ = Am − Aw, As = Am − Ax. The mixture of all other reagents except the enzyme was used as an Am. The mixture of all other reagents except the sample was used as an Aw. The mixture of all reagents was used as an Ax. The IC_50_ values were calculated using GraphPad Prism 8.0 (GraphPad Software, Inc., La Jolla, CA, USA) to evaluate the α-amylase inhibitory activity of the tested compounds.

### 2.15. Molecular Modeling Docking Study

The protein structure (PDB: 2QMJ) was downloaded from the RCSB PDB, and the ligand structures were obtained from PubChem [28,29]. Autodock tools (version: 1.5.6) and Autodock (version: 4.2.6) were used to conduct molecular docking [30]. The cubic grid box was set to 40 × 40 × 60 points with a grid spacing of 0.375 Å. Open babel was used to convert 3D SDF files to MOL2 files and PDBQT files to PDB/MOL2 files [31]. The docking poses that showed the lowest binding energies were visualized and analyzed in the UCSF chimera [32].

### 2.16. Statistical Analysis

All data were expressed as Mean ± S.D. We applied a parametric one-way analysis of variance (ANOVA) followed by the Duncan test to compare multiple groups with normal distribution. A Quantile-quantile (QQ) plot was used to understand whether data are normally distributed or skewed. For skewed data and the comparison of two groups, a nonparametric Mann–Whitney U was used to score the significance level. Pearson’s test was applied to understand the correlation between the variables since the dimension of the variables is small. All statistical analysis was performed by IBM SPSS Statistics version 25 (IBM Corporation, New York, NY, USA). The experimental results’ statistically significant difference was *p* < 0.05.

## 3. Results and Discussion

### 3.1. TPC, TFC and Yields in Different Solvent Fractions

Polyphenols are natural antioxidants widely distributed in fruits and vegetables [33]. In recent years, the screening of natural antioxidants has received increasing attention. TPC, TFC, and extraction rates of various solvent fractions from different geographic areas of celery seeds in China were evaluated in the current work, as shown in Table 1. Among the five investigated celery seed samples, samples from Northeast China had the highest total extraction yield (11.90%), and Guangxi samples obtained the lowest total extraction yield (8.23%). Based on the polarity of the extraction solvent, the contents of different solvents fractions were significantly different (*p* < 0.05). The extraction yield of DCM fractions ranged from 2.33 to 4.53%, with Shandong samples having the highest extraction yield of 4.53%. The extraction yield of nBuOH fractions was 1.75–3.10%, and the highest 3.10% extraction was obtained from Northeast samples, whereas the lowest, 1.75%, was obtained from Shandong samples. The highest 6.63% H_2_O fractions were obtained from Hubei samples, indicating that they contained more water-soluble compounds, such as polysaccharides and trace elements.

The TPC values of different fractions from the five celery seed samples ranged from 5.17 mg GAE/g to 80.17 mg GAE/g. The experimental results show that the nBuOH fractions contained much higher TPC values than the other solvent fractions, except for the Jiangsu samples. In contrast, the nBuOH and DCM fractions of Jiangsu samples had little different TPC values, 44.55 and 42.15 mg GAE/g, respectively. Among the DCM fractions, the samples from Jiangsu and Hubei contained higher TPC values of 42.15 and 31.88 mg GAE/g, respectively. Conversely, the TPC values of DCM fractions from Shandong and Guangxi samples were only 8.82 and 6.08 mg GAE/g. In addition, the H_2_O fractions contained low TPC values, which may be related to the solubility of polyphenolic compounds.

The TFC of solvent extracts from five celery seeds was evaluated by the aluminum chloride colorimetric method. Among the five celery seed samples, the Shandong samples (nBuOH fractions) contained the highest TFC values of 652.57 mg RE/g. The TFC values of nBuOH fractions were 5–30 times higher than other solvent fractions. In the DCM fractions, the highest TFC values of 42.36 mg RE/g were obtained from the Hubei samples, whereas the lowest TFC values of 19.30 mg RE/g were from the Guangxi samples. The TFC values of H_2_O fractions ranged from 5.94 to 22.49 mg RE/g, possibly due to the differences in the species and quality of flavones in the different samples.

The experimental results show that TFC and TPC values differed considerably in five samples (*p* < 0.05). This could be because they come from different geographic origins or different seasons. It is well known that plant polyphenol contents depend on several factors such as variety, location, and environmental conditions [34,35]. Similarly, significant differences were observed in total phenolic and flavonoid contents, reflecting the influence of locality with different climate conditions. For example, Sandra et al. found that the contents of the common phenolic profiles in propolis from different sources varied considerably [20]. The study by Xu et al. showed that the variety and contents of polyphenolic components in chestnuts differ significantly in different geographic regions of China [19]. The studies are also in agreement with our experimental results. In addition, no detailed studies on different geographic areas of celery seed polyphenols and flavones have been reported so far.

### 3.2. Analysis and Quantification of Main Flavones in the nBuOH Fractions of Celery Seeds from Different Geographic Areas

Table 2 shows the quantification of the six flavone glycosides (mg/g) in the nBuOH fractions from different samples by HPLC-UV. The contents of the six flavone glycosides were calculated as milligrams per gram of sample (mg/g sample). The regression equations and correlation coefficients for the six flavone standards are provided in Table S1 and Figure S1. In total, six flavone glycosides were detected in the nBuOH fractions of all five samples, but the different flavone glycosides were significantly different in all samples (*p* < 0.05). Overall, the highest flavone glycoside contents (553.01 mg/g) were observed in Shandong samples. In contrast, the Jiangsu samples contained only 154.96 mg/g. The results also indicate that the geographic region strongly influences the active ingredients contained in the plant. The highest content of graveobioside A was found in individual flavone glycosides, followed by graveobioside B and apiin. In addition, different samples showed a significant difference (*p* < 0.05) in the six flavone glycoside contents. 

As a whole, celery seed samples from all five regions of China were rich in flavones. Among the six flavone glycosides detected in this study, graveobioside A and graveobioside B were the two most predominant flavone glycosides in different celery seeds, which is consistent with the results of previous studies [18].

**Table 2:** The flavones’ structures and contents analyzed in the nBuOH fractions from different geographical areas’ celery seeds.

RT (min)	Flavones	Formula	Molecular Weight	Tandem Mass Spectrometry		Flavonoids Content in nBuOH Fractions from Different Geographic Areas Celery Seeds (mg/g)				
						Shandong Samples	Guangxi Samples	Northeast Samples	Jiangsu Samples	Hubei Samples
34.266	Graveobioside A	C_26_H_28_O_15_	580.1428	579.1334, 447.0908, 285.0381		296.68 ± 5.35 ^a,A^	276.38 ± 7.39 ^a,B^	294.95 ± 4.22 ^a,A^	87.36 ± 1.97 ^a,D^	204.72 ± 5.50 ^a,C^
35.276	Luteolin-7-O-glucoside	C_21_H_20_O_11_	448.1006	447.0925, 285.0400		26.14 ± 0.41 ^d,B^	25.25 ± 0.66 ^d,C^	29.98 ± 0.46 ^A^	8.17 ± 0.15 ^d,E^	22.82 ± 0.50 ^d,D^
37.810	Apiin	C_26_H_28_O_14_	564.1479	563.1390, 269.0452		43.08 ± 0.89 ^c,A^	40.86 ± 0.95 ^c,B^	37.03 ± 0.24 ^c,C^	10.98 ± 0.06 ^c,E^	30.64 ± 0.57 ^c,D^
38.394	Graveobioside B	C_27_H_30_O_15_	594.1585	593.1502, 299.0554		176.22 ± 3.71 ^b,A^	165.53 ± 4.55 ^b,B^	171.79 ± 1.94 ^b,A^	45.41 ± 0.11 ^b,D^	107.36 ± 2.37 ^b,C^
39.106	Apigenin-7-O-Glucoside	C_21_H_20_O_10_	432.1056	431.0979, 268.0377		7.28 ± 0.18 ^e,B^	5.64 ± 0.04 ^e,D^	6.57 ± 0.13 ^d,C^	1.89 ± 0.19 ^e,E^	9.43 ± 0.08 ^e,A^
39.952	Chrysoeriol-7-O-glucosid	C_22_H_22_O_11_	462.1162	461.1082, 299.0560		3.60 ± 0.09 ^e,A^	3.37 ± 0.06 ^e,A^	3.53 ± 0.02 ^e,A^	1.16 ± 0.40 ^e,C^	2.84 ± 0.02 ^f,B^
						553.01 ± 10.58 ^A^	517.04 ± 13.65 ^B^	543.85 ± 6.77 ^A^	154.96 ± 2.69 ^D^	377.81 ± 8.97 ^C^

Data were expressed as mean ± standard deviation (*n* = 3). Means in the same column with unlike superscripts ^(a–f)^ differ significantly (*p* < 0.05). Means in the same line with unlike superscripts ^(A–E)^ differ significantly (*p* < 0.05).

### 3.3. Antioxidant Capacities of Different Fractions from Five Celery Seed Samples

Free radicals are produced by normal biochemical reactions in the human body, which increase oxidative stress and may damage biomolecules, such as proteins and DNA [22]. Free radicals are associated with many pathological conditions, such as cancer, aging, inflammation, diabetes, ischemic heart disease, and neurodegenerative diseases. One of the therapeutic approaches to combat these diseases is searching for potential antioxidant candidates to reduce oxidative stress in the body. We firstly evaluated the antioxidant activity of different solvent fractions from celery seeds produced in different geographic areas of China by DPPH, ABTS, FRAP, CUPRAC, and Metal Chelating assays. As shown in Table 3, all fractions exhibited significant antioxidant activity.

The DPPH radical has a single electron with strong absorption at 520 nm, and its alcohol solution is purple. Antioxidants (or radical scavengers) can pair single electrons, decreasing the absorbance value of 520 nm and stopping the solution from fading [36]. Therefore, the hydrogen donating ability or the electron-donating ability of different solvent fractions can be determined utilizing the DPPH method. As shown in Table 3, the DPPH radical scavenging ability of different solvent fractions in five celery seeds produced in different geographic areas in China was tested. The results show that the DPPH radical scavenging ability of nBuOH fractions from all five samples was stronger than other fractions, which may be related to the high TPC and TFC values. Among the nBuOH fractions, all four samples showed stronger antioxidant capacity than BHT (IC_50_ = 38.70 μg/mL), except for the Jiangsu sample with IC_50_ = 44.13 μg/mL. The H_2_O fractions of all five samples showed the worst DPPH radical scavenging ability. In addition, the DCM fractions of all five samples exhibited moderate DPPH radical scavenging ability compared to the H_2_O fractions. 

The ABTS method is more reflective of highly hydrophilic antioxidants than the DPPH method [37]. As shown in Table 3, the H_2_O fractions from all five samples exhibited moderate ABTS radical scavenging. Moreover, H_2_O fractions from Shandong and Guangxi samples exhibited higher ABTS radical scavenging ability than their DCM fractions. This may be due to other active compounds in the H_2_O fractions capable of scavenging ABTS free radicals in addition to polyphenolic compounds. Therefore, some highly water-soluble compounds in the H_2_O fractions may have a better ability to scavenge ABTS radicals. In addition, the DPPH method is an organic (alcohol) and aqueous assay with an affinity for hydrophilic and hydrophobic compounds, which may lead to the ability of some highly water-soluble compounds to scavenge more ABTS radicals than DPPH radicals. Consistent with the DPPH radical scavenging ability, the nBuOH fractions of the five samples exhibited the best ABTS radical scavenging ability. The nBuOH fractions of Northeast samples showed the best ABTS radical scavenging ability (IC_50_ = 15.11 μg/mL). In contrast, the DCM fractions from Guangxi exhibited the worst ABTS radical scavenging ability (IC_50_ = 386.17 μg/mL).

The total antioxidant capacity was measured by the FRAP method. Briefly, the test method involves the reduction of Fe^3+^ to Fe^2+^ by antioxidants under acidic conditions, and the absorbance of the Fe^2+^-TPTZ complex is measured at 593 nm, which can be used as an indicator of the total antioxidant capacity [38]. Since the reaction occurs under acidic conditions, some interfering factors of endogenous origin can be suppressed. The total antioxidant capacity of the different solvent fractions from five samples ranged from 16.33 mg TE/g to 547.93 mg TE/g. Unsurprisingly, the nBuOH fractions of the five samples still exhibited the best total antioxidant capacity and were much stronger than the other fractions. Consistent with the DPPH radical scavenging, in nBuOH fractions, the four samples still showed a higher total antioxidant capacity than the positive control BHT (406.39 mg TE/g), except for the Jiangsu sample (268.33 mg TE/g). Among the DCM fractions, the total antioxidant capacity of the Jiangsu samples (117.54 mg TE/g) was significantly stronger than the other samples. In addition, the H_2_O fractions in all samples showed almost the weakest antioxidant capacity. 

The CUPRAC is a method for determining the reducing capacity of copper ions [39]. The CUPRAC method is presently widely used to determine the antioxidant capacity of dietary polyphenols and flavonoids. The test principle is the reduction of Cu^2+^ to Cu^+^ by antioxidants, and then Cu^+^ forms a stable Cu(I)-neocuproine complex with the neocuproine [40]. CUPRAC method has an advantage over the FRAP method due to faster kinetic parameters of the redox chemistry of Cu^2+^ [41]. The CUPRAC of different fractions from the five celery seed samples ranged from 22.32 mg TE/g to 465.78 mg TE/g. The Hubei n-BuOH sample exhibited the strongest CUPRAC. In each sample, the reducing capacity of copper ions was nBuOH fractions > DCM fractions > H_2_O fractions. Among the DCM fractions, the best record was still held by the Jiangsu samples (147.86 mg TE/g). In contrast, among the H_2_O fractions, the Hubei samples had the strongest antioxidant capacity by the CUPRAC method (67.00 mg TE/g).

Transition metals are considered catalysts for the initial formation of radicals. Chelating agents can inhibit the generation of free radicals by stabilizing the transition metals in the living system, reducing the damage caused by free radicals to the body [14]. Therefore, the chelating ability of transition metals is also an important method for evaluating antioxidant activity. The experimental results show that all fractions could chelate iron ions, ranging from 3.41 to 20.81 mg EDTA/g. The DCM fractions of Jiangsu samples show the most potent ability to chelate iron ions (20.81mg EDTA/g). Unsurprisingly, the H_2_O fractions of all five samples exhibited a feeble ability to chelate iron ions.

Pearson’s correlation test performed the correlation between antioxidant activity and total polyphenols and flavonoids (Table S2). High and significant positive correlations were detected between total phenolic content and antioxidant activities of DPPH, ABTS, FRAP, CUPRAC, and metal chelating. TFC was significantly correlated with ABTS, FRAP, CUPRAC, and metal chelating. Previously, many studies have shown a positive correlation between antioxidant activity and total polyphenols in plants [42]. This is also consistent with our results.

DM is a metabolic disease prevalent worldwide in people of all ages. Studies have shown that pathophysiological changes in DM may directly correlate with free radical levels [43]. In addition, free radicals have also been reported to be one of the causes of DM. The level of free radicals in the body was higher in diabetic patients than in normal people [44]. Studies have shown that antioxidant therapy can slow down complications in patients with DM [45]. Therefore, inhibiting and scavenging free radicals with natural antioxidants, such as polyphenols, may be an optimal therapy to combat the oxidative stress of DM.

Previous studies have shown that polyphenols (e.g., flavonoids, anthocyanins) are responsible for plants’ free radical collection and antioxidant activity. Polyphenolic compounds have good antioxidant and free radical scavenging activities based on the principle that one or more phenolic groups react with hydrogen donors and neutralize free radicals [46]. Currently, many studies have found celery roots, stems, and leaves have the property of scavenging hydroxyl radical and DPPH radicals but also can reduce liposome peroxidation [47]. The seeds of celery are rich in polyphenolic compounds, especially flavonoids. However, few reports on the antioxidant and free radical scavenging capacity of celery seeds were available. Our study showed that all solvent fractions of all five samples exhibited free radical scavenging and antioxidant capacity due to the high levels of polyphenolic compounds. Moreover, the nBuOH fractions show potent free radical scavenging, antioxidant, and metal ion chelating abilities due to their high TPC values. The good free radical scavenging, antioxidant, and metal ion chelating ability of celery seeds also predicts their potential as a preventive and therapeutic agent for DM.

**Table 3:** The antioxidant activities of different solvent extracts from celery seeds produced in different geographical areas in China determined by DPPH, ABTS, FRAP, CUPRAC, and Metal Chelating assays.

Code	Region	Fraction	DPPH IC_50_ (μg/mL)	ABTS IC_50_ (μg/mL)	FRAP (mg TE/g)	CUPRAC (mg TE/g)	Metal Chelating (mg EDTAE/g)
Sample 1	Shandong China	DCM	404.94 ± 14.83 ^e^	186.17 ± 3.69 ^d^	31.27 ± 2.04 ^k^	42.52 ± 0.91 ^k^	13.70 ± 0.23 ^d,e^
		nBuOH	20.49 ± 0.19 ^l^	15.49 ± 0.32 ^k^	509.93 ± 20.01 ^b^	414.32 ± 10.02 ^c^	14.11 ± 0.99 ^d^
		H_2_O	>1000 ^a^	179.43 ± 4.82 ^d^	31.02 ± 0.13 ^k^	29.82 ± 2.19 ^l^	5.71 ± 0.21 ^f^
Sample 2	Guangxi China	DCM	896.00 ± 78.14 ^d^	386.17 ± 25.18 ^a^	16.33 ± 0.90 ^n^	27.14 ± 0.06 ^l m^	16.17 ± 0.65 ^c^
		nBuOH	21.97 ± 0.81 ^k,l^	17.15 ± 0.17 ^k^	486.07 ± 9.31 ^c^	394.32 ± 9.97 ^d^	18.28 ± 0.20 ^b^
		H_2_O	>1000 ^b^	260.03 ± 13.14 ^c^	23.24 ± 0.05 ^l^	24.81 ± 0.16 ^m,n^	4.33 ± 0.43 ^g^
Sample 3	Northeast China	DCM	100.86 ± 3.40 ^g^	64.21 ± 5.62 ^g^	77.33 ± 1.38 ^h^	98.10 ± 3.98 ^h^	12.77 ± 1.06 ^e^
		nBuOH	20.27 ± 0.32 ^l^	15.11 ± 0.22 ^k^	547.93 ± 9.32 ^a^	444.78 ± 4.22 ^b^	18.33 ± 0.18 ^b^
		H_2_O	>1000 ^c^	308.17 ± 21.69 ^b^	19.57 ± 0.11 ^m^	22.32 ± 0.08 ^n^	4.08 ± 0.47 ^g^
Sample 4	Jiangsu China	DCM	87.63 ± 0.45 ^h^	40.49 ± 0.68 ^i^	117.54 ± 3.70 ^f^	147.86 ± 5.16 ^f^	20.81 ± 1.13 ^a^
		nBuOH	44.13 ± 1.44 ^i^	33.63 ± 0.85 ^j^	268.33 ± 3.41 ^e^	234.84 ± 2.46 ^e^	13.16 ± 1.06 ^d,e^
		H_2_O	823.25 ± 17.22 ^d^	141.27 ± 0.75 ^e^	44.86 ± 0.12 ^j^	56.44 ± 0.83 ^j^	3.41 ± 0.19 ^g^
Sample 5	Hubei China	DCM	109.55 ± 0.57 ^f^	44.52 ± 2.92 ^h^	100.20 ± 1.47 ^g^	116.77 ± 3.39 ^g^	13.64 ± 0.96 ^d,e^
		nBuOH	22.92 ± 0.50 ^k^	15.50 ± 1.26 ^k^	523.90 ± 3.53 ^b^	465.78 ± 3.09 ^a^	18.05 ± 0.48 ^b^
		H_2_O	512.03 ± 15.45 ^e^	117.43 ± 1.01 ^f^	53.96 ± 1.19 ^i^	67.00 ± 0.93 ^i^	3.80 ± 0.84 ^g^
VC ^A^			39.13 ± 0.93 ^j^	5.72 ± 0.10 ^l^	406.39 ± 4.65 ^d^	-	-
BHT ^A^			5.32 ± 0.11 ^m^	3.79 ± 0.04 ^l^	-	-	-

All values are reported as mean ± SD of three independent experiments. TE: Trolox equivalent; EDTAE: EDTA equivalent. ^A^ BHT and VC used as positive control of DPPH, ABTS, and FRAP. Means in the same column with unlike superscripts ^(a–n)^ differ significantly (*p* < 0.05).

### 3.4. Enzyme Inhibitory Activity of Different Fractions from Five Celery Seed Samples

α-Glucosidase hydrolyzes the α-1,4-glucosidic bond of oligosaccharides to release glucose [48]. Thus, the inhibition of α-glucosidases can delay the hydrolysis of oligosaccharides and the digestion of carbohydrates. Celery seeds from different origins showed dose-dependent inhibition of α-glucosidase, and the IC_50_ values are shown in Table 4. In the same sample, the nBuOH fractions showed the best inhibitory effect, while the DCM and H_2_O fractions were less effective. Among them, the nBuOH fractions from Hubei samples showed the strongest α-glucosidase inhibitory activity (48.79 μg/mL). Interestingly, the DCM fractions (70.24 μg/mL) of the Northeast sample showed more vigorous α-glucosidase inhibitory activity than the nBuOH fractions from the Shandong (80.57 μg/mL), Guangxi (89.20 μg/mL), and Jiangsu (90.72 μg/mL) samples. In addition, the DCM fractions of Jiangsu and Hubei samples also showed moderate α-glucosidase inhibitory activities at 235.17 and 302.10 μg/mL, respectively. Paradoxically, the H_2_O fractions of the five samples showed almost no α-glucosidase inhibitory activity. 

A-Amylase is an essential enzyme secreted by the body in the pancreas and salivary glands. A-Amylase hydrolyzes the α-1,4-glycosidic bonds within starch to generate dextrins, oligosaccharides, and monosaccharides [48]. Thus, the inhibition of α-amylase delays the digestion of starchy foods, lowing postprandial blood glucose. The inhibitory effect of α-amylase on the different fractions of celery seeds is shown in Table 4. The inhibition was significantly different from α-glucosidase. The DCM fractions exhibited higher α-amylase inhibition than nBuOH and H_2_O fractions. Moreover, the DCM fractions of the Northeastern samples showed the most excellent inhibition of α-amylase (IC_50_ = 34.69 μg/mL), which was more potent than acarbose (IC_50_ = 75.48 μg/mL), and the difference was statistically significant (*p* < 0.05). The results show a significant difference between the different fractions. In contrast to α-glucosidase, the nBuOH fractions exhibited poor α-amylase inhibitory activity. This may be related to the weak inhibition of α-amylase by the flavonoid glycosides in the nBuOH fractions. In addition, the H_2_O fractions showed almost no α-glucosidase inhibitory activity.

α-Amylase and α-glucosidases can rapidly degrade carbohydrates into monosaccharides, allowing blood glucose to rise [49]. Inhibition of α-amylase and α-glucosidases can delay the metabolism of carbohydrates and reduce the amount of glucose released by the system, thereby reducing postprandial blood glucose [50]. Studies have shown that the inhibition of α-amylase and α-glucosidase is feasible for treating type 2 DM [51]. Many active ingredients in plants, especially flavonoids, have substantial inhibitory effects on α-glucosidase and α-amylase [9,12,22,26]. These natural polyphenols showed good hypoglycemic and antioxidant abilities in vivo and in vitro [52,53]. This is also consistent with our findings. Therefore, natural α-glucosidase and α-amylase inhibitors are gradually gaining attention as promising alternatives to synthetic enzyme inhibitors (e.g., acarbose).

### 3.5. Antioxidant Activities of the Main Flavones in Celery Seeds

The antioxidant activities of the main flavones in celery seeds, that is, apigenin, luteolin, chrysoeriol, apigenin-7-O-glucoside, luteolin-7-O-glucoside, chrysoeriol-7-O-glucoside, apiin, graveobioside A, and graveobioside B were also determined by DPPH, ABTS, FRAP, CUPRAC, and metal chelating assays. As shown in Table 5, luteolin, luteolin-7-O-glucoside, and graveobioside A showed better DPPH and ABTS free radical scavenging activities. Among them, luteolin (IC_50_ = 4.65 μg/mL) exhibited the stronger DPPH radical scavenging ability than the positive control VC (IC_50_ = 5.32 μg/mL) and BHT (IC_50_ = 39.13 μg/mL). In addition, luteolin-7-O-glucoside (DPPH IC_50_ = 9.28 μg/mL) and graveobioside A (DPPH IC_50_ = 12.47 μg/mL) also exhibited a stronger DPPH radical scavenging ability than positive control BHT (DPPH IC_50_ = 39.13 μg/mL). The DPPH radical scavenging ability of apigenin, chrysoeriol, and their glycosides was poor. However, they had moderate ABTS radical scavenging ability. In the FRAP and CUPRAC assays, luteolin, luteolin-7-O-glucoside, and graveobioside A also showed the best antioxidant effect than other flavonoids in celery seeds. In contrast, luteolin, apigenin, and chrysoeriol showed considerable metal chelation of 88.44, 54.20, and 36.49 mg EDTA/g, respectively. Unexpectedly, the graveobioside A showed the best metal ion chelation (89. 98 mg EDTA/g). Different from flavone disaccharides, three flavone monosaccharide in celery seed hardly exhibited metal ion chelating effects.

**Figure 2:**
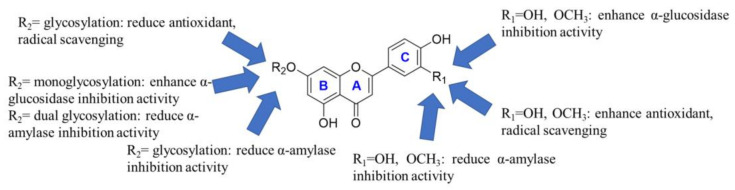
Structure–activity relationship analysis of flavonoids in celery seeds.

Structure–activity relationship analysis revealed that the hydroxyl group at the 3’-position in the C ring of flavones contributes to their antioxidant and radical scavenging effects (see Figure 2). In addition, the methylation or the hydrogenation in the 3’-position in the C ring of flavones significantly reduces their antioxidant and radical scavenging effect. However, methylation at the 3′-position in the C ring of flavonoids is still beneficial for enhancing their antioxidant and free radical scavenging abilities. At the same time, glycosylation of the hydroxyl group at the 7-positions in the B ring of flavones would also reduce the antioxidant capacity and radical scavenging, consistent with previous reports [52]. The monosaccharide of flavones showed stronger DPPH radical scavenging ability than their disaccharides, while the opposite was for ABTS radical scavenging ability. Metal ion chelation experiments revealed that the 7-position hydroxyl glycosylation of flavone B ring is not favorable for its metal ion chelation, and the monoglycosylation is particularly unfavorable. However, the double glycosylation of luteolin is an exception.

**Table 5:** The antioxidant, α-glucosidase inhibitory, and α-amylase inhibitory activities of the main flavonoids in celery seeds.

No.	Compounds	DPPH IC_50_ (μM)	ABTS IC_50_ (μg/mL)	FRAP (mg TE/g)	CUPRAC (mg TE/g)	Metal Chelating (mg EDTAE/g)	α-Glucosidase IC_50_ (μM)	α-Amylase IC_50_ (mM)
1	Graveobioside A	12.47 ± 0.08 ^h^	6.88 ± 0.05 ^h^	882.04 ± 0.43 ^b^	887.17 ± 8.95 ^c^	89.98 ± 0.42 ^a^	104.31 ± 5.75 ^b^	N.A.
2	Graveobioside B	3266.33 ± 365.08 ^c^	29.43 ± 1.33 ^e^	166.87 ± 2.97 ^g^	328.10 ± 14.10 ^e^	21.32 ± 1.51 ^e^	103.96 ± 1.85 ^b^	N.A.
3	Apiin	7686.33 ± 200.18 ^a^	1055.33 ± 24.68 ^a^	5.97 ± 1.49 ^j^	25.26 ± 0.18 ^g^	17.15 ± 1.37 ^f^	198.70 ± 14.91 ^a^	N.A.
4	Luteolin-7-O-glucoside	9.28 ± 0.06 ^i^	9.41 ± 0.42 ^g^	830.71 ± 8.52 ^c^	955.05 ± 3.64 ^b^	8.51 ± 0.21 ^g^	49.87 ± 2.85 ^d^	N.A.
5	Chrysoeriol-7-O-glucosid	848.23 ± 143.61 ^e^	46.64 ± 1.24 ^d^	203.35 ± 6.33 ^f^	495.51 ± 6.64 ^d^	8.22 ± 0.84 ^g^	39.79 ± 1.05 ^e^	1.86 ± 0.02 ^b^
6	Apigenin-7-O-Glucoside	4885.33 ± 56.01 ^b^	461.00 ± 11.04 ^b^	25.90 ± 0.49 ^i^	33.45 ± 0.51 ^g^	5.69 ± 0.19 ^h^	77.47 ± 4.84 ^c^	1.74 ± 0.04 ^c^
7	Luteolin	4.65 ± 0.16 ^j^	4.58 ± 0.02 ^i,j^	1025.12 ± 10.78 ^a^	1502.63 ± 15.92 ^a^	88.44 ±0.64 ^b^	58.83 ± 2.97 ^d^	2.11 ± 0.01 ^a^
8	Chrysoeriol	580.10 ± 35.54 ^f^	16.69 ± 0.55 ^f^	337.96 ± 0.88 ^e^	947.43 ± 5.72 ^b^	36.49 ± 0.22 ^d^	40.49 ± 0.53 ^e^	1.31 ± 0.03 ^d^
9	Apigenin	1292.00 ± 31.00 ^d^	223.63 ± 10.35 ^c^	59.41 ± 0.24 ^h^	76.44 ± 1.83 ^f^	54.20 ± 1.28 ^c^	79.98 ± 0.85 ^c^	0.86 ± 0.01 ^e^
10	BHT ^A^	39.13 ± 0.93 ^g^	5.72 ± 0.10 ^h,i^	406.39 ± 4.65 ^d^	N.	N.	N.	N.
11	VC ^A^	5.32 ± 0.11 ^j^	3.79 ± 0.04 ^j^	N.	N.	N.	N.	N.
12	Acarbose ^B^	N.	N.	N.	N.	N.	0.023 ± 0.00 ^f^	0.12 ± 0.00 ^f^

All values are reported as mean ± SD of three independent experiments. ^A^ BHT and VC used as positive control of DPPH, ABTS, and FRAP. ^B^ Acarbose used as positive control of enzyme inhibition test. TE: Trolox equivalent; EDTAE: EDTA equivalent. N. indicates not test. N.A. indicates no available. Means in the same column with unlike superscripts ^(a–j)^ differ significantly (*p* < 0.05).

### 3.6. α-Glucosidase and α-Amylase Inhibition Activity of the Main Flavones in Celery Seeds

Three kinds of aglycons, that is, chrysoeriol, luteolin, and apigenin, directly inhibited α-glucosidase activity in vitro (Table 5). Their IC_50_ values were 36.49 μM, 88.44 μM, and 54.20 μM, respectively. The IC_50_ of acarbose was 0.023 μM, which agrees with previous reports [27]. Surprisingly, the α-glucosidase inhibitory activities of three flavone-7-O-glucosides were superior to their aglycones and disaccharides. This may be related to their binding mode to α-glucosidase. The α-glucosidase inhibition assays revealed that the hydroxyl group and methoxy at the 3’-position of the flavone C ring are favorable for their binding to α-glucosidase (see Figure 2). At the same time, monoglycosylation of the hydroxyl group at the 7-positions in the B ring of flavones would also enhance the α-glucosidase inhibition activity. However, the dual glycosylation of the hydroxyl group at the 7-positions in the B ring of flavones would reduce the α-glucosidase inhibition activity. Consistent with previous reports, we also found that flavones were less able to inhibit α-amylase [12]. In contrast, the methoxy and the hydroxyl group at the 3’-position of the flavone C ring were prejudiced to α-Amylase inhibitory activity. Moreover, the 7-position glycosylation of the flavone B ring significantly attenuated the α-amylase inhibitory activity. As previously reported, the α-amylase inhibitory activity of apiin was weak, with an IC_50_ of 1084 μg/mL [12].

Previous reports have shown that flavonoids have α-glucosidase and α-amylase inhibiting activities and can affect starch digestion by forming complexes. It can be used as a dietary supplement for natural α-amylase and α-glucosidase inhibitors to prevent or treat DM [12,13,21,22]. For example, luteolin strongly inhibits α-amylase and α-glucosidase, which may reduce the risk of type 2 DM [21]. In addition, flavonoids can also form complexes with starch through hydrophobic interaction, affecting starch digestion and reducing starch’s digestibility [13]. All of this evidence suggests that natural flavonoids are an excellent preventive or therapeutic agent for DM. In short, celery seeds are expected to be a valuable new resource for regulating postprandial blood glucose levels.

### 3.7. Molecular Modeling Docking

**Figure 3:**
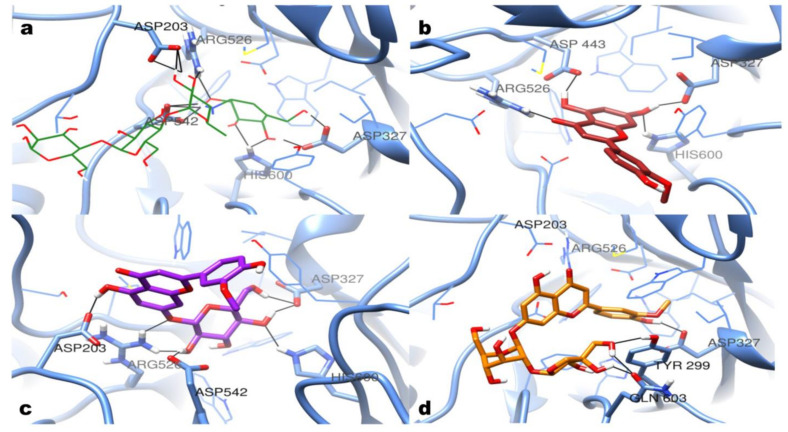
Molecular docking results. The predicted binding mode of (**a**) acarbose (green, wire); (**b**) chrysoeriol (brown, stick); (**c**) chrysoeriol-7-O-glucoside (purple, stick); (**d**) graveobioside B (orange, stick).

As the results show, none of the identified compounds or extractions displayed better inhibitory activities than acarbose. Monoglycosides showed slightly better inhibitory activities than the corresponding flavone aglycones, while the di-glycosides showed poor activities. All nine identified compounds have been docked into the active site of α-glucosidase (PDB: 2QMJ). As shown in Figure 3, 12 hydrogen bonds were found in the binding site of acarbose with ASP203, ASP327, ARG526, ASP542, and HIS600. According to the docking results, chrysoeriol could form four hydrogen bonds with ASP327, ASP443, ARG526, and HIS600. Chrysoeriol-7-O-glucoside (thermopsoside) could form seven hydrogen bonds with ASP203, ASP327, ARG526, ASP542, and HIS600. Unlike them, graveobioside B exhibited different hydrogen bond interactions with TYR299 and GLN603. It indicated that the di-glycosides were not fitting well in the active site of α-glucosidase because of the introduction of the apiose.

## 4. Conclusions

In this study, different fractions and major flavones from celery seeds produced in different geographical regions of China were systematically evaluated by different antioxidant systems, α-glucosidase inhibition, and α-amylase inhibition activity assays. Significant differences (*p* < 0.05) were found between different samples and fractions in TPC, TFC, DPPH, ABTS, FRAP, CUPRAC, and metal chelating values. The TPC and TFC values of nBuOH fractions showed the highest values among all solvent fractions in the same sample, indicating that nBuOH fractions had the highest polyphenol and flavonoid contents, which is consistent with the antioxidant activity results. In addition, nBuOH fractions from Hubei samples showed the strongest glucosidase inhibition ability. In contrast, the strongest amylase inhibitory activity was obtained by the DCM fractions from the Northeast samples. 

This study also assessed the contents of flavones in the nBuOH fractions of celery seeds from different geographical regions of China. The results show that the Shandong samples had the highest content of the six flavones. In vitro antioxidants show that six flavonoid glycosides and their aglycones have suitable antioxidant activities, especially luteolin and its glycosides. Chrysoeriol-7-O-glucoside shows potent α-glucosidase inhibitory activity. Molecular docking indicated that chrysoeriol-7-O-glucoside has a high affinity for α-glucosidase and thus displays potential α-glucosidase inhibitory activity.

In conclusion, this study is the first to systematically evaluate antioxidant, α-glucosidase inhibitory, and α-amylase inhibitory activities in different fractions of celery seeds from different geographical regions. It provides a scientific basis for the antioxidant and hypoglycemic applications of celery seeds. In addition, the findings also imply that celery seed extracts and the flavonoid components therein have the potential as natural antioxidants to prevent oxidative deterioration in foods, pharmaceuticals, cosmetics, and other products.

## Figures and Tables

**Table 1:** TPC, TFC, and extraction yields of five celery seed samples with different solvents.

Code	Region	Fraction	TPC (mg GAE/g)	TFC (mg RE/g)	Yields (%)
Sample 1	Shandong China	DCM	8.82 ± 0.39 ^k^	26.40 ± 1.23 ^g,h^	4.53
		nBuOH	71.90 ± 0.90 ^c^	652.57 ± 12.14 ^a^	1.75
		H_2_O	7.79 ± 0.82 ^k^	22.49 ± 0.76 ^h^	4.20
					10.48
Sample 2	Guangxi China	DCM	6.08 ± 0.11 ^l,m^	19.30 ± 0.54 ^h,i^	3.55
		nBuOH	65.17 ± 0.12 ^d^	589.31 ± 3.94 ^c^	1.85
		H_2_O	7.24 ± 0.42 ^k,l^	13.08 ± 0.79 ^i,j^	2.83
					8.23
Sample 3	Northeast China	DCM	24.42 ± 0.40 ^h^	34.10 ± 1.16 ^f,g^	3.68
		nBuOH	80.17 ± 0.57 ^a^	632.21 ± 6.56 ^b^	3.10
		H_2_O	5.17 ± 0.97 ^m^	8.83 ± 0.12 ^j^	3.07
					11.90
Sample 4	Jiangsu China	DCM	42.15 ± 2.56 ^f^	39.82 ± 1.20 ^f^	2.33
		nBuOH	44.55 ± 0.29 ^e^	201.92 ± 0.08 ^e^	2.76
		H_2_O	10.79 ± 0.38 ^j^	5.94 ± 0.10 ^j^	5.00
					10.09
Sample 5	Hubei China	DCM	31.88 ± 0.14 ^g^	42.36 ± 2.56 ^f^	3.33
		nBuOH	74.71 ± 0.93 ^b^	467.20 ± 10.74 ^d^	1.82
		H_2_O	14.47 ± 1.46 ^i^	11.00 ± 0.18 ^j^	6.63
					10.48

All values are reported as mean ± SD of three independent experiments. GAE: Gallic acid equivalent; RE: Rutin equivalent. The yield was calculated as % yield = (weight of extract/initial weight of dry sample) × 100; Means in the same column with unlike superscripts ^(a–m)^ differ significantly (*p* < 0.05).

**Table 4:** The α-glucosidase and α-amylase inhibitory activity of different solvent extracts from celery seeds produced in different geographical areas in China.

Code	Region	Fraction	α-Glucosidase IC_50_ (μg/mL)	α-Amylase IC_50_ (μg/mL)
Sample 1	Shandong province	DCM	N.A.	349.03 ± 2.43 ^f^
		nBuOH	80.57 ± 3.96 ^d^	749.73 ± 15.51 ^b^
		H_2_O	N.A.	N.A.
Sample 2	Guangxi province	DCM	-	95.96 ± 4.43 ^g,h^
		nBuOH	89.20 ± 3.45 ^c^	626.93 ± 26.04 ^d^
		H_2_O	N.A.	N.A.
Sample 3	Northeast China	DCM	70.24 ± 4.69 ^e^	34.69 ± 1.62 ^i^
		nBuOH	57.00 ± 3.93 ^f^	391.07 ± 12.92 ^e^
		H_2_O	N.A.	N.A.
Sample 4	Jiangsu province	DCM	235.17 ± 0.51 ^b^	119.43 ± 2.32 ^g^
		nBuOH	90.72 ± 2.06 ^c^	984.27 ± 26.67 ^a^
		H_2_O	N.A.	N.A.
Sample 5	Hubei province	DCM	302.10 ± 1.82 ^a^	103.23 ±3.04 ^g^
		nBuOH	48.79 ± 2.65 ^g^	675.13 ± 11.27 ^c^
		H_2_O	N.A.	N.A.
Acarbose ^B^			0.015 ± 0.001 ^h^	75.48 ± 2.50 ^h^

All values are reported as mean ± SD of three independent experiments. N.A. indicates that no data were available. ^B^ Acarbose used as positive control of enzyme inhibition test. Means in the same column with unlike superscripts ^(a–i)^ differ significantly (*p* < 0.05).

## Data Availability

Data are contained within the article.

## Supplementary Materials

The following supporting information can be downloaded at: https://www.mdpi.com/article/10.3390/antiox11081542/s1, Figure S1: Chromatogram of the standards and nBuOH fractions from five samples; Figure S2: The standard curve of six flavonoid glycosides and TPC, TFC, FRAP, CUPRAC and metal chelating; Table S1: The detection wavelengths, retention time, regressive equations, and correlation coefficient of six flavonoid glycosides; Table S2: Pearson’s correlation coefficients (r) for the relationships between antioxidant assays, phenolic contents, and flavonoid contents.
