# Supplementary material for: Antioxidant Capacities and Enzymatic Inhibitory Effects of Different Solvent Fractions and Major Flavones from Celery Seeds Produced in Different Geographic Areas in China
Source: Antioxidants (Basel). 2022 Aug 9;11(8):1542. doi: 10.3390/antiox11081542 (PMC9404946; doi:10.3390/antiox11081542)
Supplement: Supplementary file 1 [file antioxidants-11-01542-s001.zip › antioxidants-1835344-supplementary.pdf]

Table S1. The detection wavelengths, retention time, regressive equations and correlation coefficient of six flavonoid glycosides.

No.	Flavonoids	Detection wavelengths	Retention time (min)	Regressive equations [a]	R^2
1	Graveobioside A	270 nm	34.266	y = 33.603x + 825.73	0.9999
2	Luteolin-7-O-glucoside	270 nm	35.276	y = 51.599x - 1251.8	0.9999
3	Apiin	270 nm	37.810	y = 60.778x + 537.85	0.9999
4	Graveobioside B	270 nm	38.394	y = 39.306x + 2397.9	0.9999
5	Apigenin-7-O-Glucoside	270 nm	39.106	y = 84.896x + 41.423	0.9999
6	Chrysoeriol-7-O-glucosid	270 nm	39.952	y = 46.219x + 278.37	0.9999

[a] The y value is the peak area of flavonoid glycosides and the x is the concentration of samples (ng/mL).

Table S2. Pearson's correlation coefficients (r) for the relationships between antioxidant assays, phenolic contents and flavonoid contents.

Variables	TPC	TFC	DPPH	ABTS	FRAP	CUPRAC
TFC	0.922 **					
DPPH	0.621 *	0.462				
ABTS	0.796 **	0.609 *	0.602 *			
FRAP	0.970 **	0.978 **	0.529 *	0.695 *		
CUPRAC	0.983 **	0.958 **	0.571 *	0.732 **	0.996 **	
Metal Chelating	0.701 **	0.552 *	0.638 *	0.488	0.602 *	0.639 *

** Significant correlation with $p < 0.01$; * Significant correlation with $p < 0.05$.

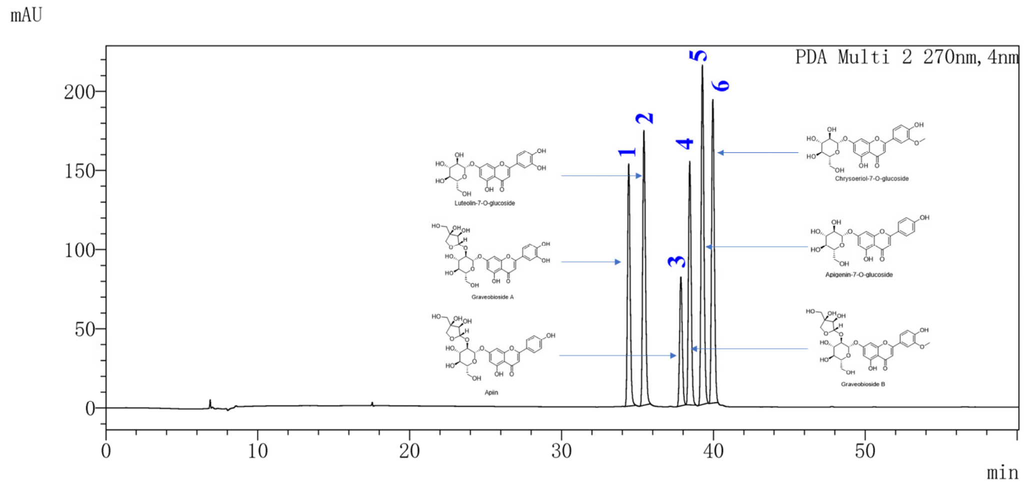

(a) Chromatograms of the standards.

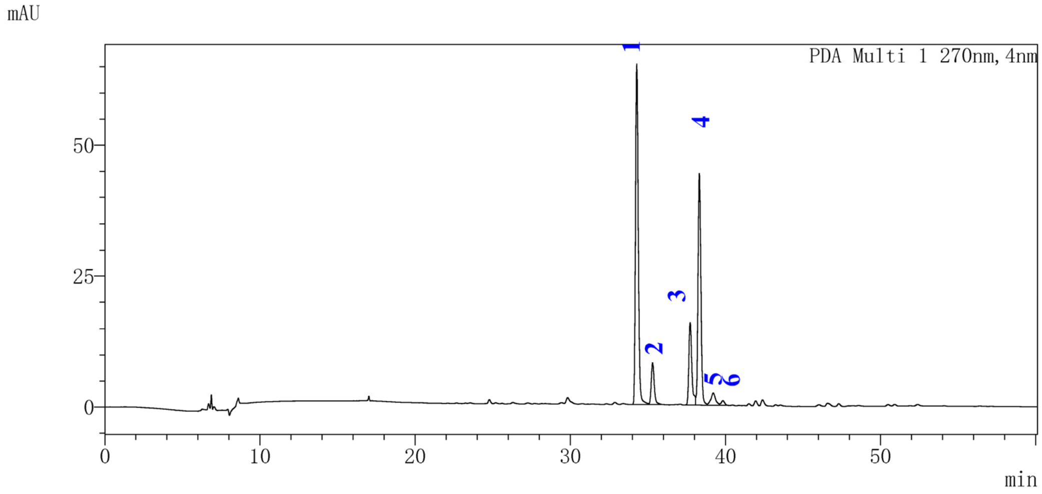

(b) Chromatograms of the n-butanol fractions from Shandong, China samples.

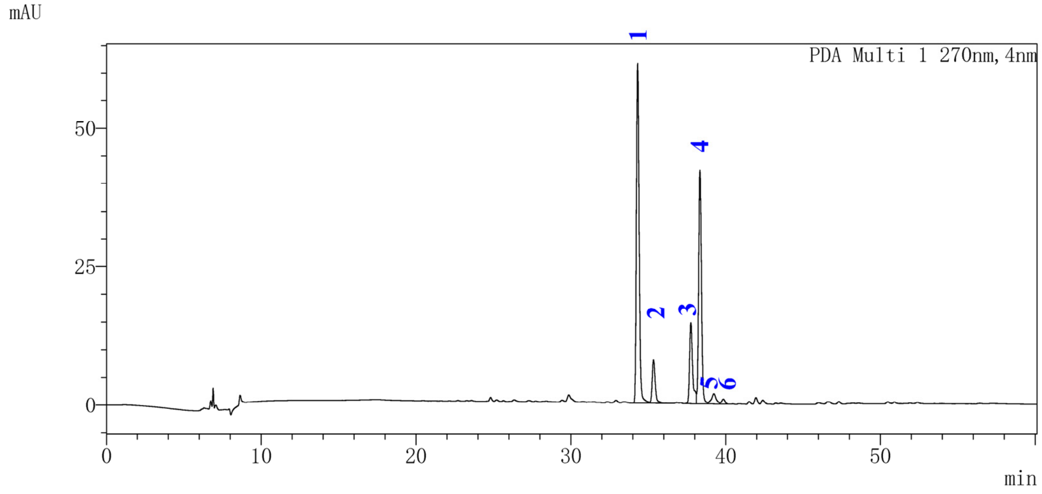

(c) Chromatograms of the n-butanol fractions from Guangxi, China samples.

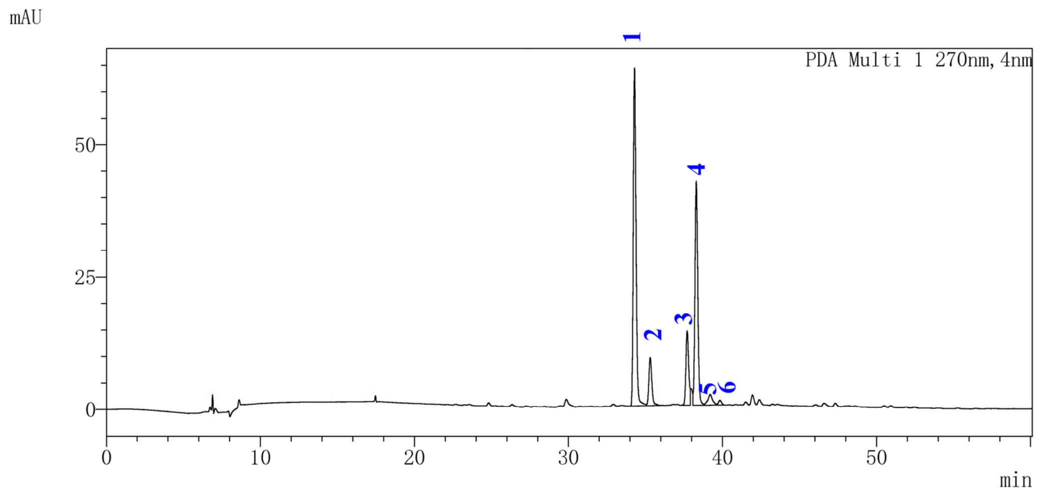

(d) Chromatograms of the n-butanol fractions from Northeast, China samples.

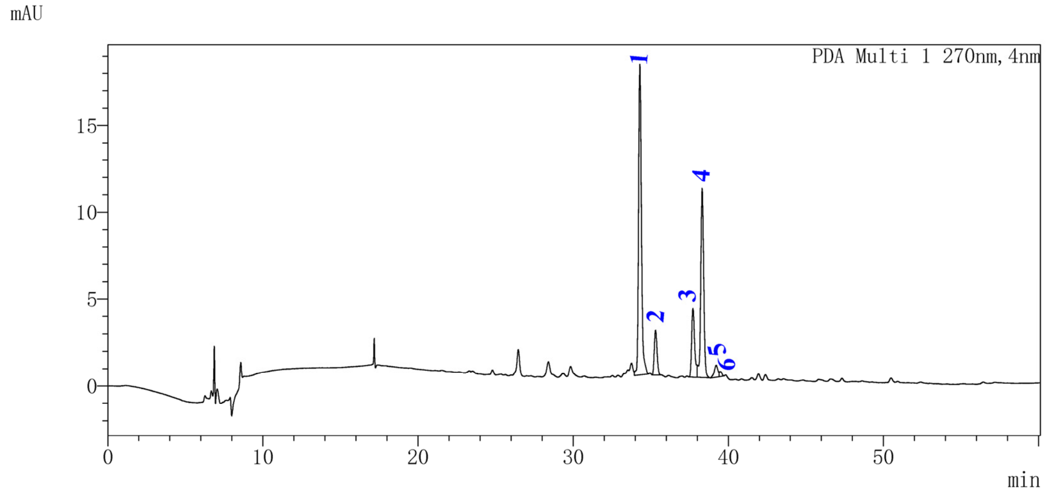

(e) Chromatograms of the n-butanol fractions from Jiangsu, China samples.

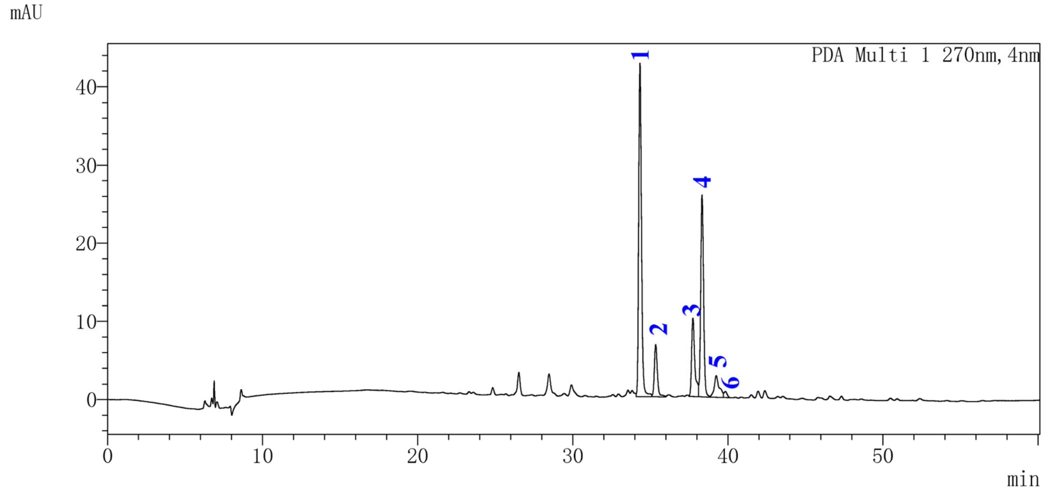

(f) Chromatograms of the n-butanol fractions from Hubei, China samples.

Figure S1. Chromatogram of the standards and n-butanol fractions from five samples.

1: Graveobioside A; 2: Luteolin-7-O-glucoside; 3: Apiin; 4: Graveobioside B; 5: Apigenin-7-O-glucoside; 6:

Chrysoeriol-7-O-glucoside.

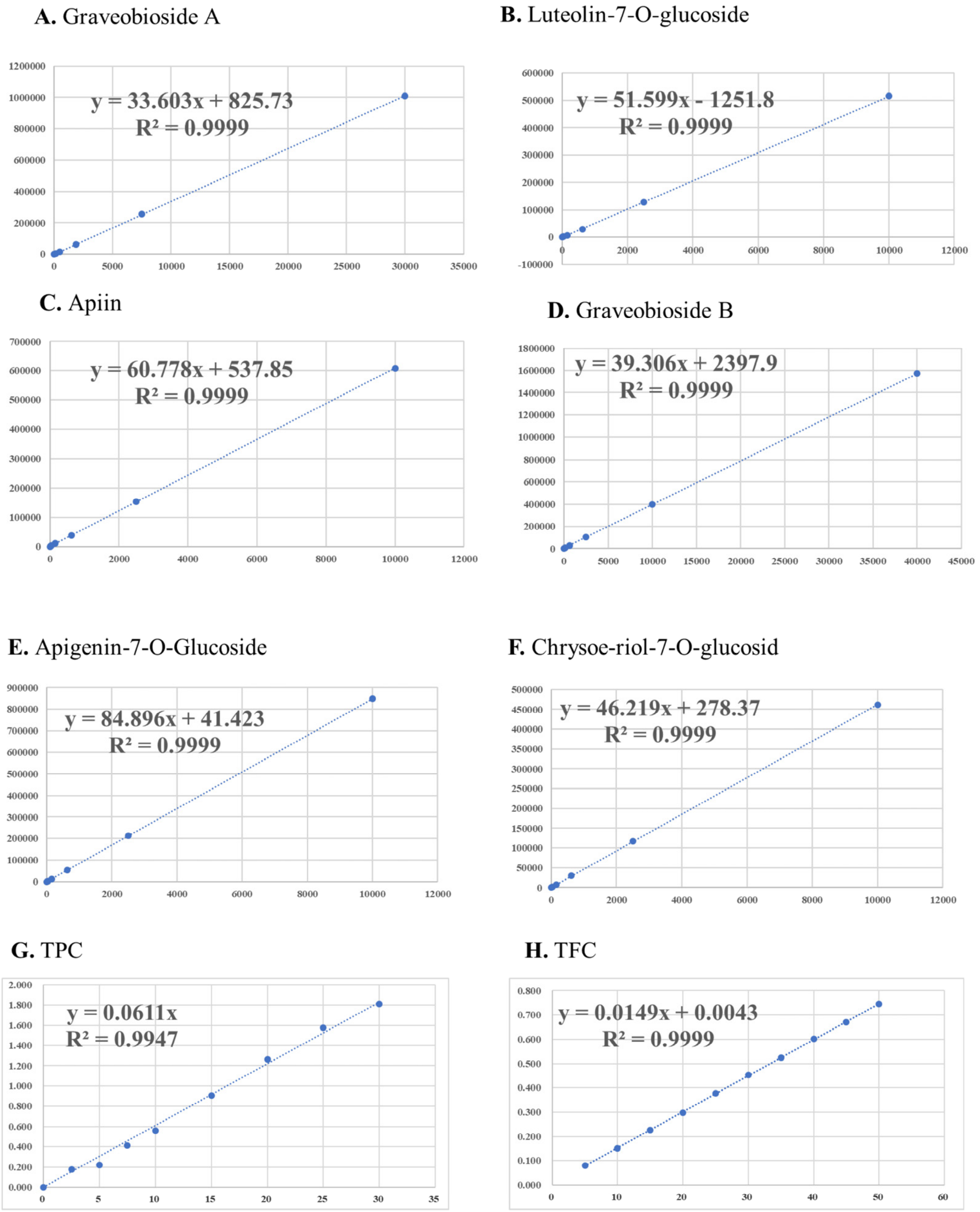
A. Graveobioside A
y = 33.603x + 825.73
R² = 0.9999
B. Luteolin-7-O-glucoside
y = 51.599x - 1251.8
R² = 0.9999
C. Apiin
y = 60.778x + 537.85
R² = 0.9999
D. Graveobioside B
y = 39.306x + 2397.9
R² = 0.9999
E. Apigenin-7-O-Glucoside
y = 84.896x + 41.423
R² = 0.9999
F. Chrysoe-riol-7-O-glucosid
y = 46.219x + 278.37
R² = 0.9999
G. TPC
y = 0.0611x
R² = 0.9947
H. TFC
y = 0.0149x + 0.0043
R² = 0.9999

I. FRAP

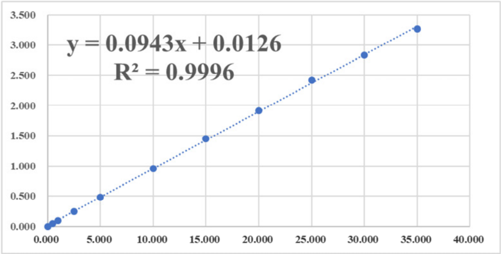

J. CUPRAC

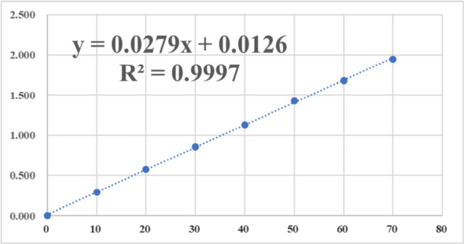

K. Metal Chelating

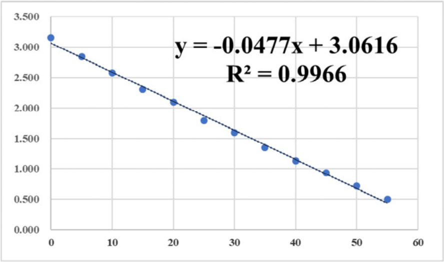

Figure S2. The standard curve of six flavonoid glycosides and TPC, TFC, FRAP, CUPRAC and metal chelating.
